# Effect of Environmental Drivers on Functional Traits of Salvadora Population in the Semi‐Arid Regions: A Case Study From Division Sahiwal Pakistan

**DOI:** 10.1002/ece3.71938

**Published:** 2025-09-02

**Authors:** Shaheena Umbreen, Naila Mukhtar, Nidaa Harun, Sajid Ali

**Affiliations:** ^1^ Department of Botany, Faculty of Life Sciences University of Okara Okara Pakistan; ^2^ International Maize and Wheat Improvement Center (CIMMYT) Islamabad Pakistan; ^3^ Department of Agriculture Hazara University Mansehra Pakistan

**Keywords:** distribution, Division Sahiwal, edaphic factors, functional traits, habitat types, Pakistan, *S. oleoides*, *S. persica*, Salvadora, semi‐arid lowlands

## Abstract

Salvadora faces a significant threat of being in decline in semi‐arid regions. This study investigates the distribution status of *Salvadora* species in semi‐arid habitats, moreover examines how habitat types, climatic conditions and soil variability influence plant's functional traits and distribution. The study was organized in the semi‐arid lowlands of the Sahiwal Division, Pakistan. Field surveys were conducted from 2021 to 2023 across 51 sites comprised of four types of habitats, i.e., archaeological sites, graveyards, roadsides, and railway lines. Principal Component Analysis (PCA) and Canonical Correspondence Analysis (CCA) were applied to examine the impact of habitat types and environmental variables on Salvadora distribution. Two species of Salvadora, i.e., *Salvadora persica* Linn and *Salvadora oleoides* Decne, were identified taxonomically in the study area. *S. persica* was found to be more abundant than *S*. *oleoides*. These results recommend that *S. persica* was more dominant in most sites except for Sahiwal, where both species had similar densities. The number of tree trunks, tree height, and leaf size, leaf biomass are some of the dominant traits that were influenced by habitat variability. Other factors like temperature, precipitation, th soil's pH and moisture levels play important roles in species distribution within these habitats. Despite Salvadora notable economic and ecological importance, its ecological situation is critical because of overexploitation, climate change, and habitat destruction. To ensure that Salvadora continues to exist and perform its ecological functions in its natural habitat, protecting and managing strategies need to be planned and enforced.

## Graphical abstract

This research aimed to study the distribution status of *Salvadora* species in the semi‐arid lowlands of the Sahiwal Division, Pakistan, along with the impact of habitat type on its functional traits and the effect of climatic and edaphic variables on its distribution. Field surveys were conducted from 2021 to 2023 across 51 sites comprising four types of habitats. These results indicate a predominance of *S. persica* in comparison to S. oleiodes. Moreover, it was observed that features such as the number of tree trunks, tree height, and leaf pattern are greatly influenced by different types of habitats. Along with other factors like temperature and precipitation, soil properties like pH levels and moisture content have a significant impact on the distribution of species within these habitats.

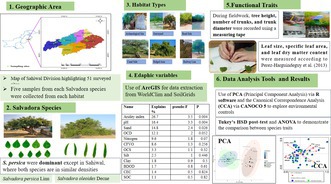

## 1 Introduction

This study analyzing the spatial distribution of native tree species and their associated risks is vital in understanding how to minimize biodiversity loss and how to manage critical components of biodiversity, especially in the face of global climatic change (Pautasso et al. 2010; Brundu et al. 2020). The existence of native trees is crucial for fostering ecosystem and biodiversity conservation as it is controlled by the various factors that are both abiotic, such as climate, soil properties, and biotic relationships (Bayat et al. 2021). Several recent studies underscores the importance of documenting species distribution patterns of native trees for effective conservation (Velazco et al. 2020; McNellie et al. 2020; Muscatello et al. 2021). A regional study conducted in South Asia also provided fundamental information about the native tree species diversity and the geographic patterns of their occurrence with respect to elevation, which necessitates attention for conservation(Chaudhary et al. 2022; Ma et al. 2023). Threats to native tree populations are multifaceted, involving both natural and anthropogenic factors, including climate change, habitat conversion, overexploitation, fire, and overgrazing, which have been quantified for their impact on species vulnerability (Koulelis et al. 2023). These threats can lead to reductions in species abundance and even local extinctions, significantly altering ecosystem functions the distribution of numerous native tree species provide insights into how environmental factors shape species distributions and their ecological niches (Arshad et al. 2022; Waheed et al. 2023). These studies highlight the necessity of comprehensive data and robust analytical methods to form conservation policies for native tree species and their associated ecosystems (Fremout et al. 2020).

These threats can lead to reductions in species abundance and even local extinctions, significantly altering ecosystem functions (Leclerc et al. 2020). Studies analyzing the distribution of numerous native tree species provide insights into how environmental factors shape species distributions and their ecological niches (Arshad et al. 2022; Waheed et al. 2023). These studies highlight the necessity of comprehensive data and robust analytical methods to inform conservation policies for native tree species and their associated ecosystems (Fremout et al. 2020). Understanding how environmental factors affect the distribution and functional features of tree species is critical for addressing the reduction in native tree populations, which has important ecological and conservation consequences (Sapsford et al. 2020). Environmental variables significantly influence the distribution patterns and ecological niches of tree species (Maharjan et al. 2021). *Salvadora* species exhibit ecological adaptability and may be found in a variety of environments, including dry scrublands and damaged urban borders (Khan et al. 2015; Ali et al. 2020; Iqbal et al. 2023). Functional traits, including growth, survival, and reproduction, have a strong connection to environmental factors, offering useful insights into species adaptation and resilience in changing environments (Kühn et al. 2021). Thus, investigating functional features in connection with environmental factors not only improves our knowledge of ecological dynamics, but it also informs actual conservation and management methods (Volaire et al. 2020; Higham et al. 2021). The *Salvadora* genus, which belongs to the Salvadoraceae family, consists of small trees or shrubs that are mostly found in subtropical and tropical parts of Africa and Asia (Korejo et al. 2010; Latef et al. 2023). This genus is very adaptable to a variety of environments, including salty areas, wetlands, desert flood plains, and grassy savannahs (Bhandari et al. 2021). *Salvadora* species are ecologically valuable because of their capacity to flourish in a variety of adverse environmental circumstances (Falasca et al. 2015). The various parts of this plant are used in traditional medicine to cure different diseases, adding to its economic and cultural relevance in many communities (Kumar et al. 2012; Ahmad and Rajagopal 2013). The loss of habitat, climate change, and overexploitation pose serious challenges to Salvadora despite its ecological and economic significance, especially in semi‐arid locations (Korejo et al. 2010; Nafees et al. 2019; Iqbal et al. 2022). Their numbers have noticeably decreased as a result, calling for immediate conservation and sustainable management measures. Thus, the current study set out (1) to evaluate the *Salvadora* species' current distribution status in the semi‐arid lowlands region, (2) to investigate the species' habitat type and functional traits, and (3) explore the distinct effects of edaphic and climatic variables on the distribution of Salvadora in the study area.

## 2 Materials and Methods

### 2.1 Study Area

**FIGURE 1:**
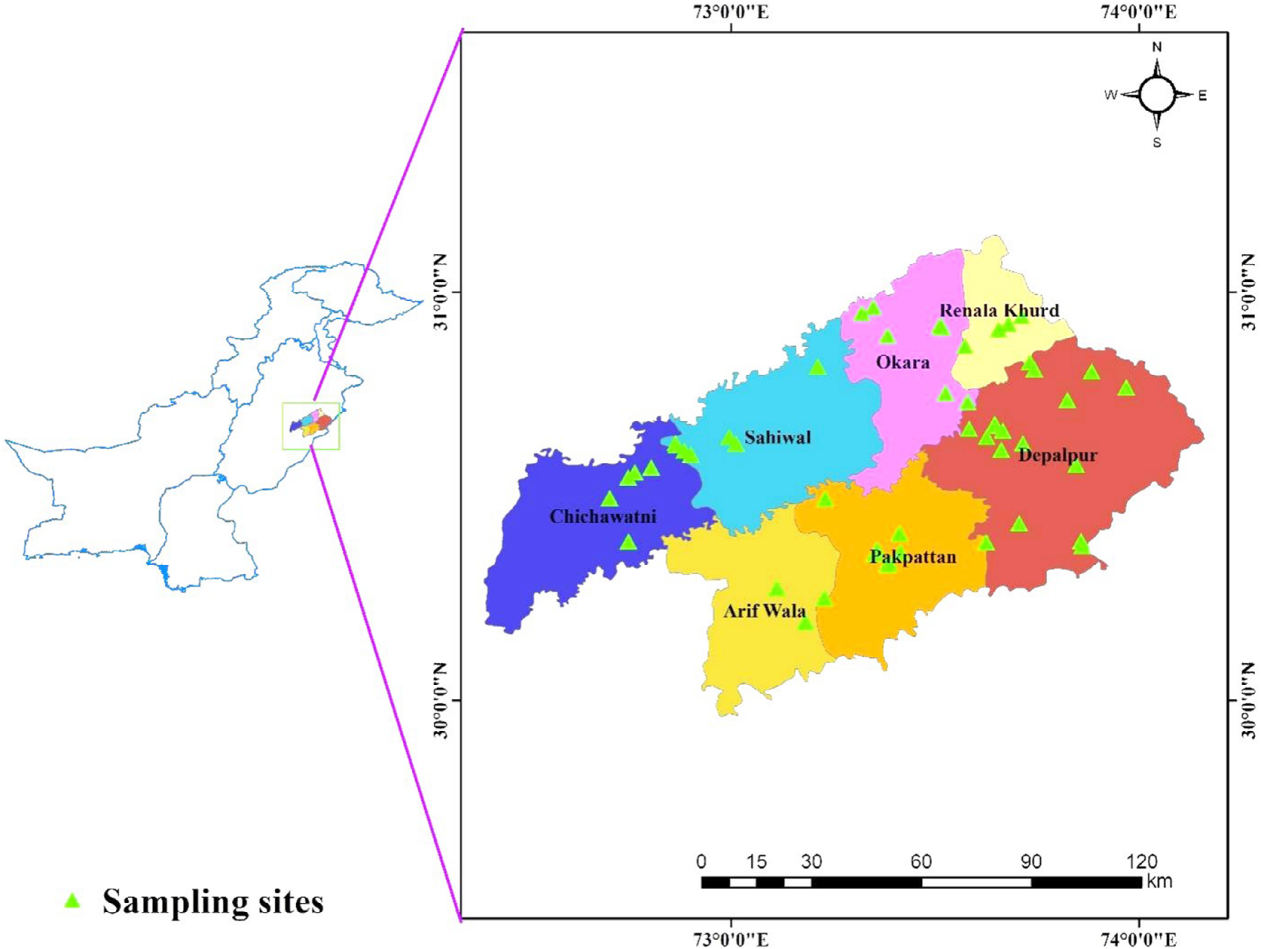
Map showing the study area and sampling locations of the Salvadora population in the semi‐arid region of Pakistan.

Sahiwal division is situated in the semi‐arid zone of central to southern Punjab, Pakistan and is renowned for its highly productive agricultural lands. This land significantly contributes to the local economy and also elevate the per capita income compared to other areas of Punjab. The region possesses a diverse landscape comprising irrigated plains, agricultural fields, canal networks, rugged hilly zones, forests, and plantations, creating a varied environment conducive to human settlement. Geographically, the research site is bounded by Sahiwal, Pakpattan, and Okara districts, situated between latitudes 29°25′12″ N to 31°28′16″ N and longitudes 71°58′34″ E to 74°43′25″ E (Figure 1). The topography of the Sahiwal Division is predominantly flat and fertile/productive, with the River Sutlej to the south and the River Chenab to the west contributing to its fertile plains. The climate is characterized by a semi‐arid environment, featuring hot and dry summers from April to October, with peak temperatures in May, June, and July, and mild winters.

### 2.2 Field Surveys and Data Collection

**FIGURE 2:**
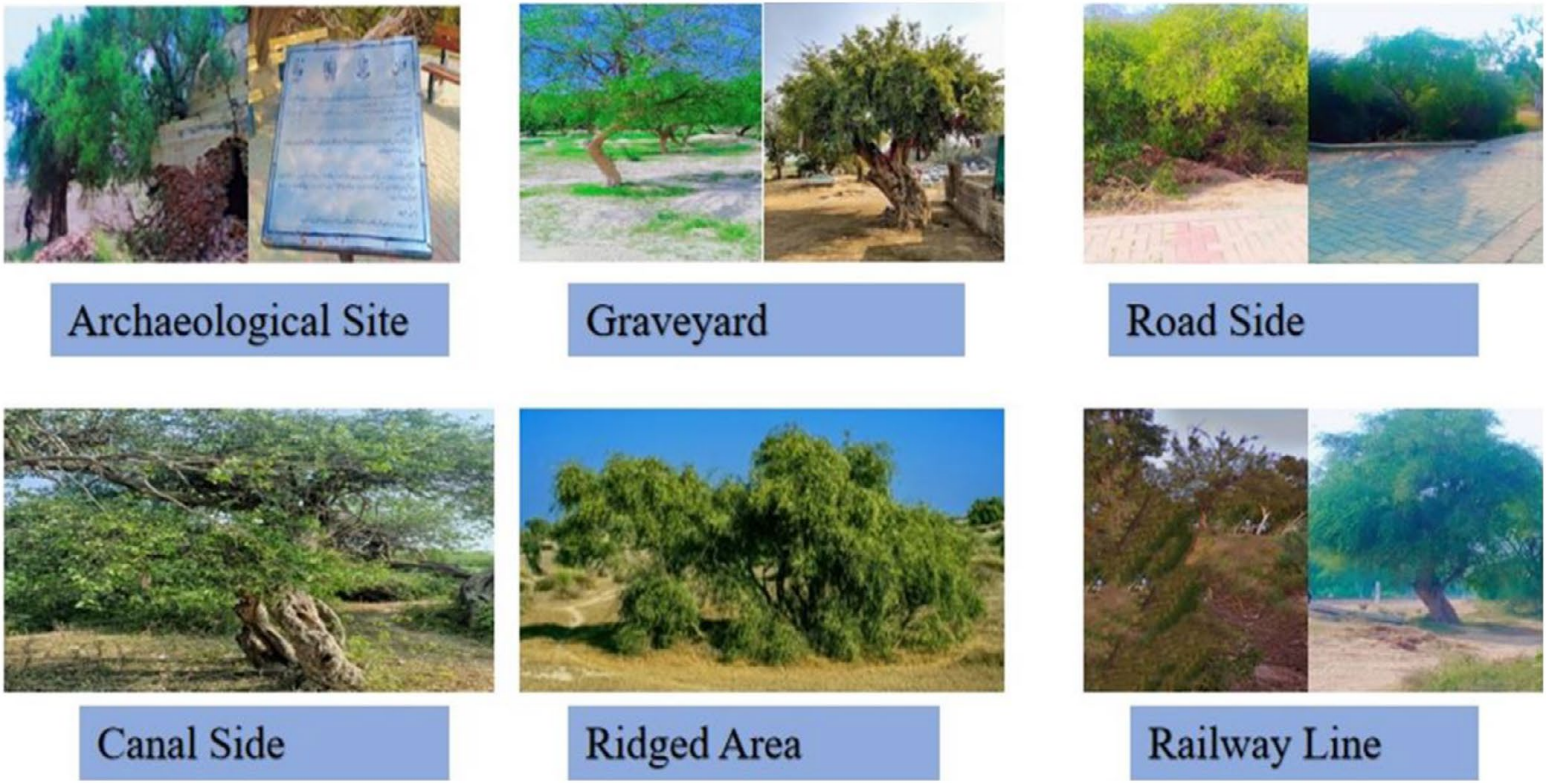
Some pectoral glimpses of the presence of Salvadora in diverse habitat types during the survey across Sahiwal.

This study follows the stratified random sampling. Site selection was conducted independently of any prior knowledge of site characteristics, reducing the risk of selection bias. Field surveys were conducted from 2021 to 2023 to document the *Salvadora* genus distribution in Pakistan's semi‐arid region. During field surveys, plant specimens were collected, photographed, pressed, and dried, and subsequently mounted on herbarium sheets conforming to international standards. Specimen identification was conducted using the online resource Flora of Pakistan (http://www.efloras.org/; Accessed September 21, 2021). Taxonomic verification was performed using Plants of the World Online (https://powo.science.kew.org/; Accessed October 10, 2022). Geographical coordinates, including elevation, latitude, and longitude, were recorded for each location using a Garmin eTrex Global Positioning System (GPS) device. Given the presence of Salvadora in diverse habitat types, a total of 51 sites were selected from multiple habitat types, including archaeological sites, graveyards, roadsides, and railway lines (Figure 2). The number of Salvadora plants was counted at each sampling site across different habitat types. During fieldwork, tree height, number of trunks, and trunk diameter were recorded using a measuring tape. Five samples from each *Salvadora* species were collected from each habitat for the measurement of leaf functional traits. Leaves were collected in polythene bags for further analysis. In the laboratory, leaf size (LS), specific leaf area (SLA), and leaf dry matter content (LDMC) were measured to assess the leaf traits comprehensively. Leaf size, specific leaf area, and leaf dry matter content were measured according to Perez‐Harguindeguy et al. (2013).

### 2.3 Edaphic Variables

The 51 sampled sites were georeferenced for mapping the distribution of Salvadora. As part of our study, we compiled a comprehensive dataset of 19 bioclimatic variables with a spatial resolution of 30 arc sec from WorldClim (version 2.1) (www.worldclim.org, accessed March 22, 2023). Additionally, we integrated 10 edaphic variables from SoilGrids (https://soilgrids.org/, accessed March 22, 2023) (Table S1). Climatic and edaphic data from the 51 locations were extracted using ArcGIS for further analysis of the impact of climatic and edaphic variables on the distribution of *Salvadora*. Since the study area is situated in irrigated plains, elevation, slope, and aspect data were not mainly focussed in this analysis. This decision was made because these factors are less variable and do not significantly influence the habitats in the irrigated plains region.

### 2.4 Data Analysis

The data on the distribution of *Salvadora* species were compiled into an Excel spreadsheet in 2013 for subsequent statistical analysis. An analysis of the ecological characteristics of the given species of Salvadora and their habitats showed that a Principal Component Analysis (PCA) method using the “vegan” package on R 4.0.0 (Oksanen 2010) was efficient in exploring the behavioral patterns among the principal species traits. This further assisted in the identification of the trait interventions among the species. A comparison of the parameters of native and non‐native species using ANOVA was conducted with the aim of finding out to what extent various traits are displayed by both species. A Tukey's HSD posttest analysis assisted in the multivariate assessment of comparisons, hence, a better understanding of the extent and nature of the differences (Haq, Lone, et al. 2023; Haq, Rashid, et al. 2023; Waheed and Arshad 2024). To assess climatic and edaphic controls on the distribution of Salvadora, Canonical Correspondence Analysis (CCA) was conducted utilizing CANOCO 5 software (Šmilauer and Lepš 2014). By illuminating the factors influencing the habitat preferences of Salvadora, this multivariate statistical approach provided insightful information about the relationships between environmental parameters and species distribution patterns (Haq, Lone, et al. 2023; Haq, Rashid, et al. 2023).

## 3 Results

### 3.1 Distribution Patterns of Salvadora

**FIGURE 3A,B:**
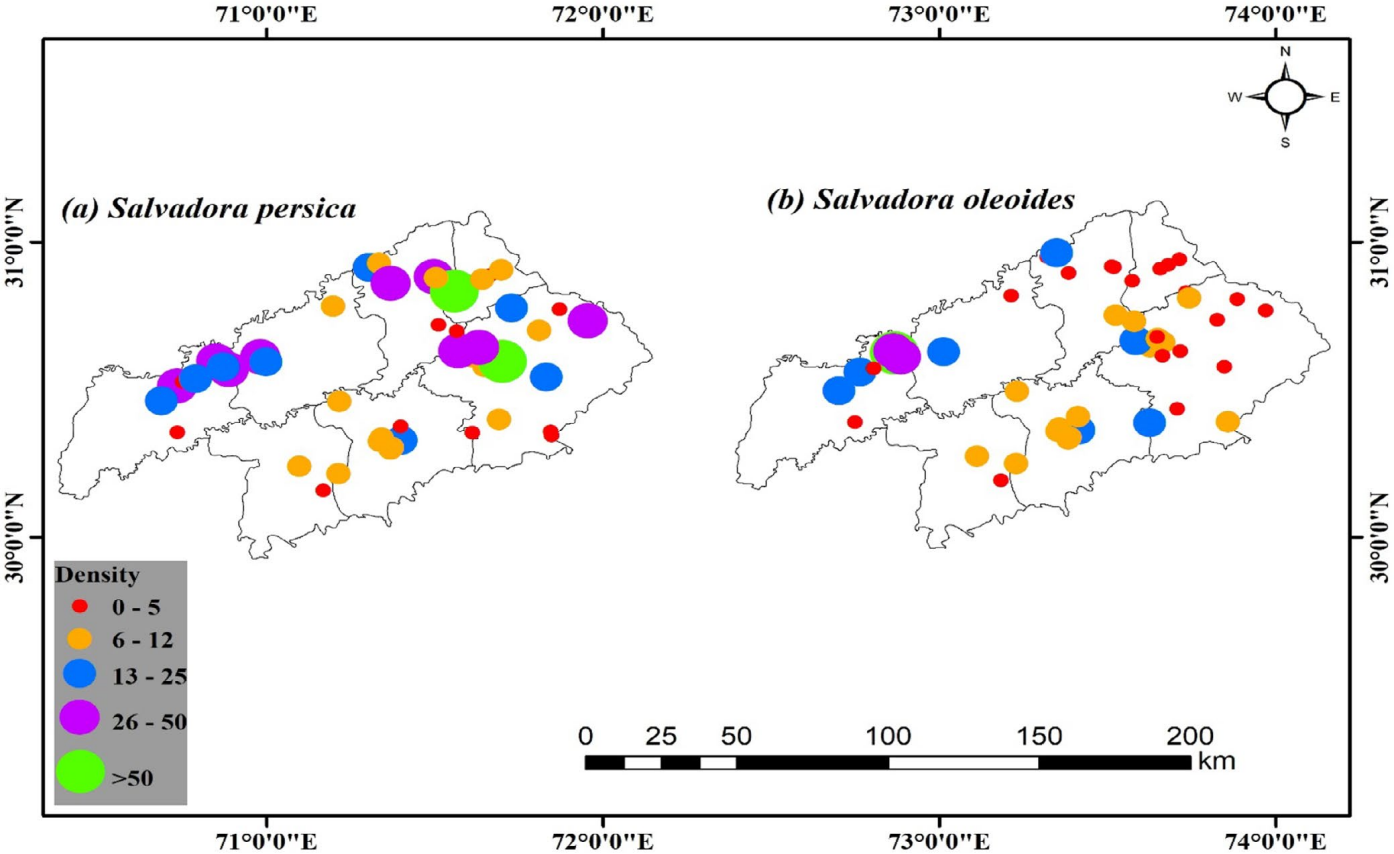
Population status and distribution pattern of genus *Salvadora* in semi‐arid lowlands.

In the semi‐arid lowlands of Division Sahiwal, researchers have identified two species that belong to the *Salvadora* genus: *Salvadora persica* and *Salvadora oleoides*. These species, which are indigenous to tropical and subtropical regions, are recognized for their medicinal, culinary, and cultural significance. In the Okara region, the average density of *S. persica* was recorded at 23 plants/m^2^, whereas *S. oleoides* was considerably rarer, with an average density of merely 1.5 plants/m^2^. A comparable pattern was observed in Depalpur, where *S. persica* exhibited an average density of 15.06 plants/m^2^, while in contrast to *S. oleoides*, which had an average density of 3 plants/m^2^, as depicted in Figure 3a,b.

Depalpur exhibited an average density of 7.33 plants/m^2^ for *S. persica* , with no *S. oleoides* recorded. Sahiwal showed a more balanced distribution, with *S. persica* having an average density of 26.13 and *S. oleoides* slightly higher at 27.25 plants/m^2^. Chichawatni had an average density of 15.33 for *S. persica* and 3.33 plants/m^2^ for *S. oleoides*. In Pakpattan, the average densities were 7.62 for *S. persica* and 4 plants/m^2^ for *S. oleoides*. Lastly, Arifwala recorded an average density of 7 for *S. persica* and 1.5 plants/m^2^ for *S. oleoides*. These results indicate a predominance of *S. persica* in most tehsils, except for Sahiwal, where both species showed comparable densities, suggesting localized factors may influence the distribution and density of these genera in the semi‐arid lowlands (Figure 3a,b).

### 3.2 Impact of Habitat Type on Functional Traits of Salvadora

**FIGURE 4:**
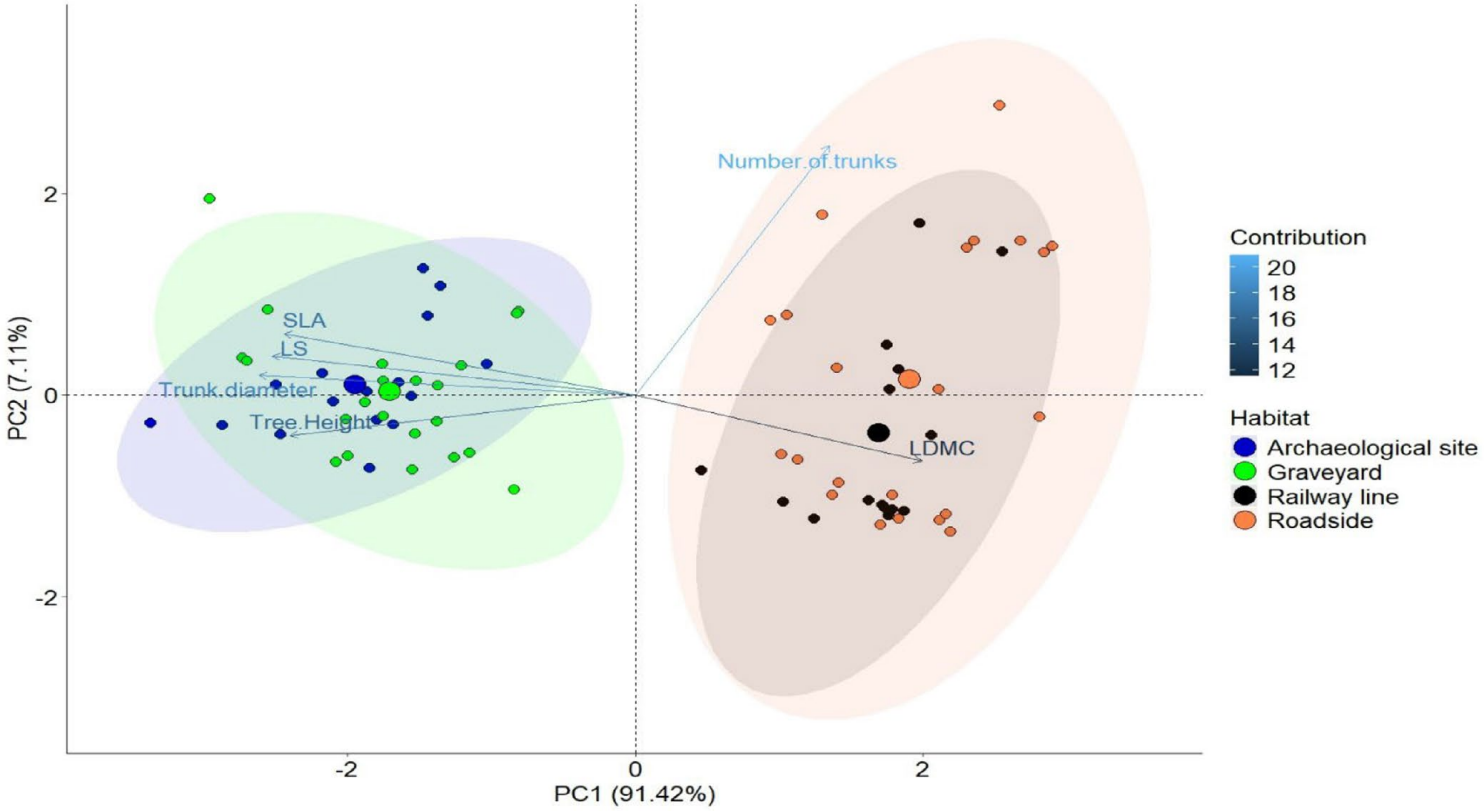
Principal Component Analysis showing the trait variation in different habitat types for Salvadora in semi‐arid regions.

The Principal Component Analysis PCA revealed that the first two principal components account for 91.4% (PC1) and 7.11% (PC2) of the variance (Figure 4). PC1 is characterized by high Leaf Dry Matter Content (LDMC) and the number of trunks on the positive axis, and high Leaf Size (LS), Specific Leaf Area (SLA), tree height, and tree diameter on the negative axis. This component is nearly aligned with the roadside and railway line habitats. Salvadora individuals' positive and negative loadings on PC1 suggest a positive association with graveyard and archaeological site habitats. PC2 is oriented with the highest SLA and LS on the positive side and the lowest SLA and LS on the negative side, indicating that leaf traits are more adapted to roadside and railway line habitats.

### 3.3 Traits Variations in Species

**FIGURE 5:**
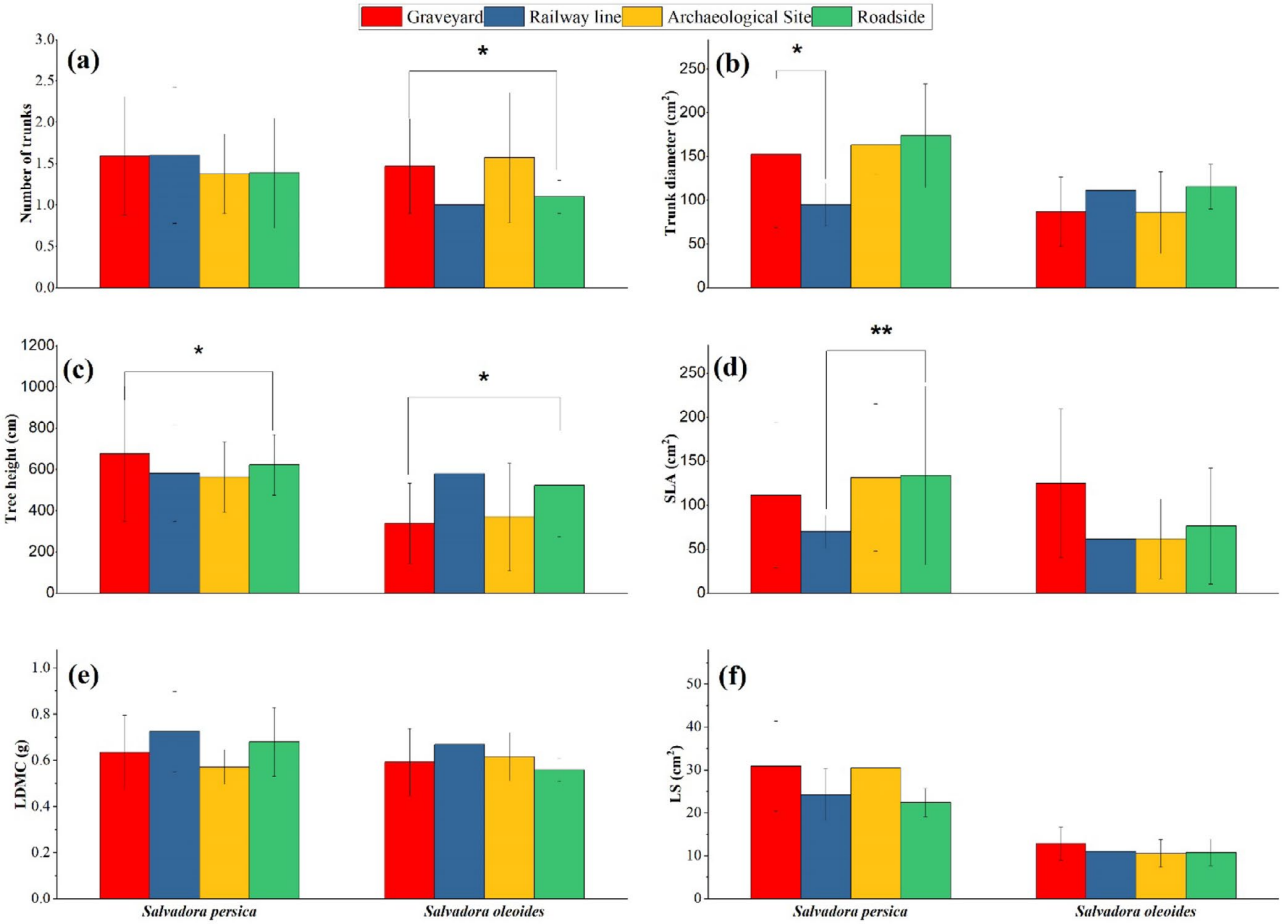
Bar chart showing the difference in various functional traits between *Salvadora persica* Linn. and *Salvadora oleoides* Decne in different habitat types of the semi‐arid region of Punjab, Pakistan.

An ANOVA followed by Tukey's post hoc test assessed the differences in various functional traits of *S. persica* and *S. oleoides* across different habitats. For *S. persica* , the trunk count ranged from 1 to 5, with trunk diameters varying from 94.80 ± 11.00 cm^2^ along railway lines to 173.81 ± 17.85 cm^2^ along roadsides. Tree heights ranged from 562.99 ± 85.21 cm at archaeological sites to 675.83 ± 54.64 cm in graveyards. Specific Leaf Area (SLA) varied from 70.02 ± 8.35 cm^2^ along railway lines to 133.53 ± 30.63 cm^2^ along roadsides, while Leaf Dry Matter Content (LDMC) ranged from 0.571 ± 0.038 g at archaeological sites to 0.723 ± 0.078 g along railway lines. Leaf Size (LS) ranged from 24.20 ± 2.71 cm^2^ along railway lines to 30.86 ± 1.74 cm^2^ in graveyards (Figure 5). For *S. oleoides*, the trunk count ranged from 1 to 4, with trunk diameters varying from 85.71 ± 17.62 cm^2^ at archaeological sites to 115.35 ± 12.82 cm^2^ along roadsides. Tree heights ranged from 370.11 ± 98.53 cm at archaeological sites to 579.12 cm along railway lines. SLA varied from 61.3 cm^2^ along railway lines to 124.71 ± 16.89 cm^2^ in graveyards, and LDMC ranged from 0.558 ± 0.025 g along roadsides to 0.668 g along railway lines. LS ranged from 10.57 ± 1.21 cm^2^ at archaeological sites to 12.84 ± 0.77 cm^2^ in graveyards (Figure 5). Significant habitat‐specific differences were observed in the measured functional traits for *S. persica* and *S. oleoides*.

**FIGURE 6:**
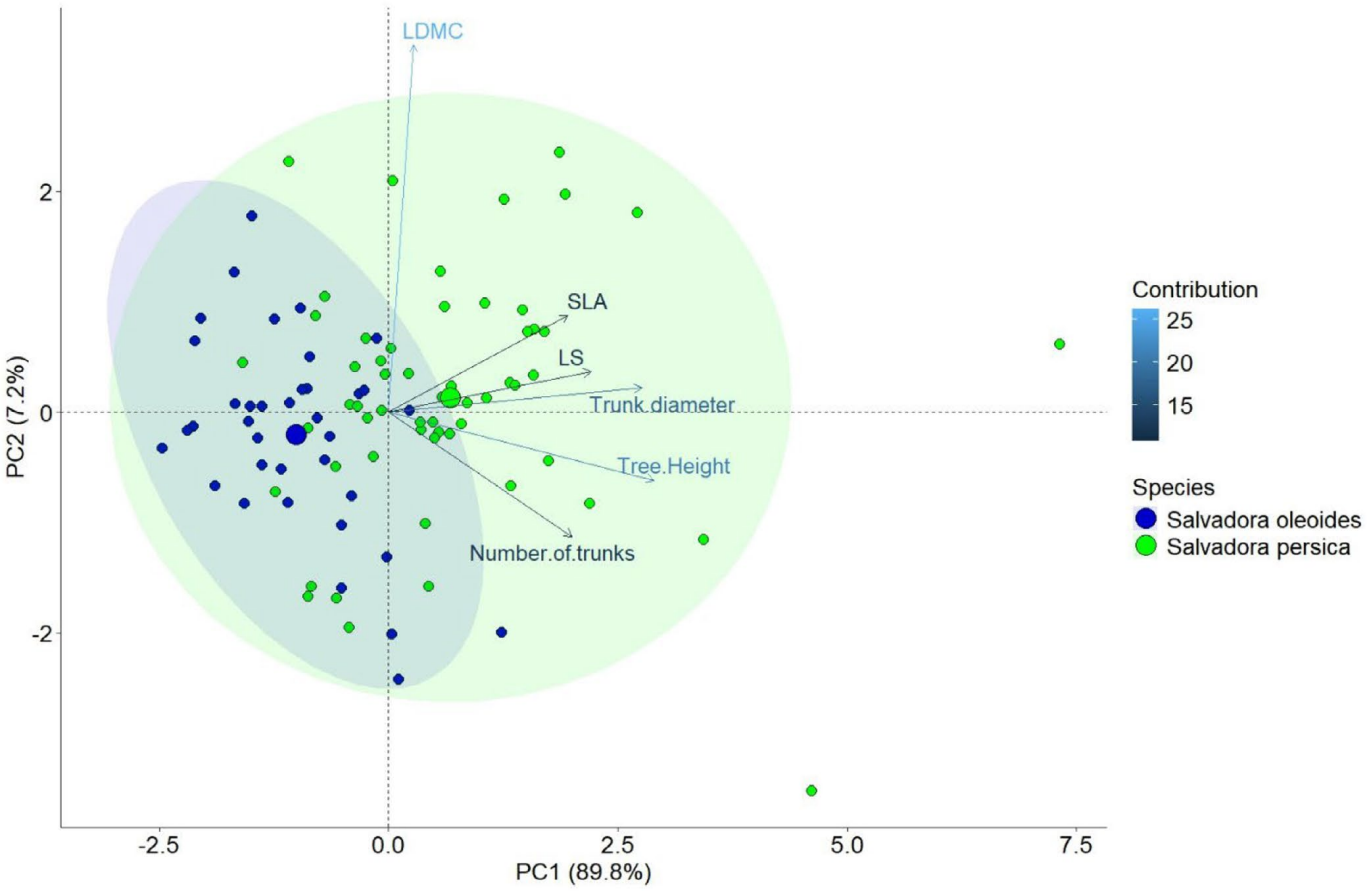
Principal Component Analysis showing the trait variation in functional traits of *Salvadora* species in semi‐arid regions.

The Principal Component Analysis (PCA) revealed distinct trait variations for both *S. persica* and *S. oleoides*. For *S. persica* , PCA1 explained 89.8% of the total variation, while PCA2 explained 7.2% (Figure 6). Notably, the number of trunks, tree height, and trunk diameter exhibited positive loadings on PCA1 and negative loadings on PCA2. Conversely, Specific Leaf Area (SLA), Leaf Size (LS), and Leaf Dry Matter Content (LDMC) showed positive loadings on PCA2 and negative loadings on PCA1. In the case of *S. persica* , the species clustered positively on both PCA1 and PCA2 axes. Conversely, *S. oleoides* exhibited negative clustering on PCA1 and positive clustering on PCA2. These distinct patterns highlight species‐specific trait variations and their responses to different environmental conditions, as elucidated by PCA.

### 3.4 Impact of Climatic Variables on the Distribution of Salvadora

**FIGURE 7:**
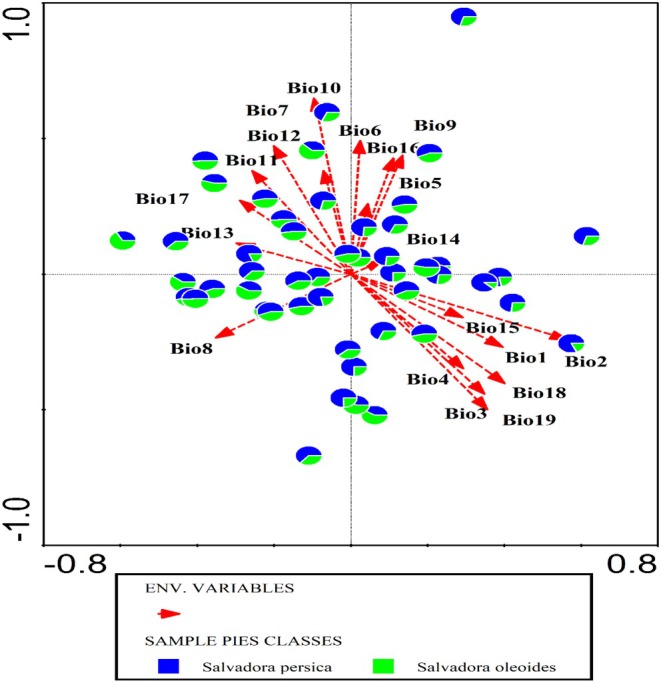
Canonical correspondence analysis (CCA) showing the impact of climatic variables on the distribution of *Salvadora* species in semi‐arid regions.

A canonical correspondence analysis (CCA) was conducted to investigate the impact of climatic variables on the distribution of the genus *Salvadora*. The total variation explained by the model was 0.01319, with the explanatory variables accounting for 38.1% of this variation (Figure 6). After adjustment, the explained variation was reduced to 5.2%. The analysis yielded eigenvalues for the first four axes as follows: Axis 1 (0.0016), Axis 2 (0.0014), Axis 3 (0.0007), and Axis 4 (0.0004) (Table 1). The cumulative explained variation by these axes was 11.87%, 22.34%, 27.67%, and 30.48%, respectively. The pseudo‐canonical correlations for these axes were moderately high, with values of 0.7547 for Axis 1, 0.6466 for Axis 2, 0.7143 for Axis 3, and 0.7167 for Axis 4. The cumulative explained fitted variation also increased across the axes, reaching 31.18%, 58.67%, 72.67%, and 80.05%. Permutation tests on all axes resulted in a pseudo‐*F* value of 1.2 with a *p*‐value of 0.17, indicating that the model's explanatory power was not statistically significant at the conventional 0.05 level. Further analysis of the simple term effects of individual climatic variables revealed significant contributions from several variables. Bio19 (Precipitation of Coldest Quarter) explained 27.7% of the variation with a pseudo‐*F* value of 4 and a *p*‐value of 0.002. Bio3 (Isothermality) explained 16.8% (pseudo‐*F* = 3.5, *p* = 0.002), Bio1 (Annual Mean Temperature) 10.8% (pseudo‐*F* = 3.5, *p* = 0.002), and Bio2 (Mean Diurnal Range) 9.4% (pseudo‐*F* = 3.5, *p* = 0.002) (Table 2). Other significant variables included Bio10 (Mean Temperature of Warmest Quarter), Bio4 (Temperature Seasonality), Bio12 (Annual Precipitation), Bio18 (Precipitation of Warmest Quarter), and Bio16 (Precipitation of Wettest Quarter), all with *p*‐values less than 0.05. CCA indicated that climatic variables collectively have a substantial impact on the distribution of Salvadora; individual variables such as Bio19, Bio3, Bio1, and Bio2 were particularly influential. However, the overall model did not achieve statistical significance in permutation tests, suggesting that other unaccounted factors may also play a significant role in influencing Salvadora distribution (Figure 7).

**TABLE 1:** Results of CCA analysis of climatic and edaphic variables impacting the distribution of Salvadora in semi‐arid region.

Variables	Statistics	Eigenvalues	Explained variation (cumulative)	Pseudo‐canonical correlation	Explained fitted variation (cumulative)
Climatic variables	Axis 1	0.0016	11.87	0.7547	31.18
Edaphic variables	Axis 1	0.0013	9.9	0.6896	38.79
Climatic variables	Axis 2	0.0014	22.34	0.6466	58.67
Edaphic variables	Axis 2	0.0008	15.66	0.5652	61.37
Climatic variables	Axis 3	0.0007	27.67	0.7143	72.67
Edaphic variables	Axis 3	0.0004	18.98	0.4799	74.4
Climatic variables	Axis 4	0.0004	30.48	0.7167	80.05
Edaphic variables	Axis 4	0.0003	21.17	0.4835	82.97

**TABLE 2:** Effect of each climatic variable on the distribution of Salvadora in the semi‐arid region.

Name	Explains %	pseudo*‐F*	*p*
Bio19	27.7	4	0.002
Bio3	16.8	3.5	0.002
Bio1	10.8	3.5	0.002
Bio2	9.4	3.5	0.002
Bio10	8.2	3.3	0.008
Bio4	3.3	3.2	0.01
Bio12	3	3.2	0.012
Bio18	2.8	3.1	0.014
Bio16	2.7	3	0.012
Bio7	2.5	2.8	0.012
Bio11	2.4	2.7	0.014
Bio9	2.2	2.4	0.034
Bio6	1.5	2.4	0.034
Bio17	1.5	2	0.052
Bio5	1.3	2	0.05
Bio15	1.2	1.8	0.094
Bio14	1	1.6	0.138
Bio13	0.9	1.4	0.206
Bio8	0.8	1.1	0.274

### 3.5 Impact of Edaphic Variables on Salvadora Distribution

**FIGURE 8:**
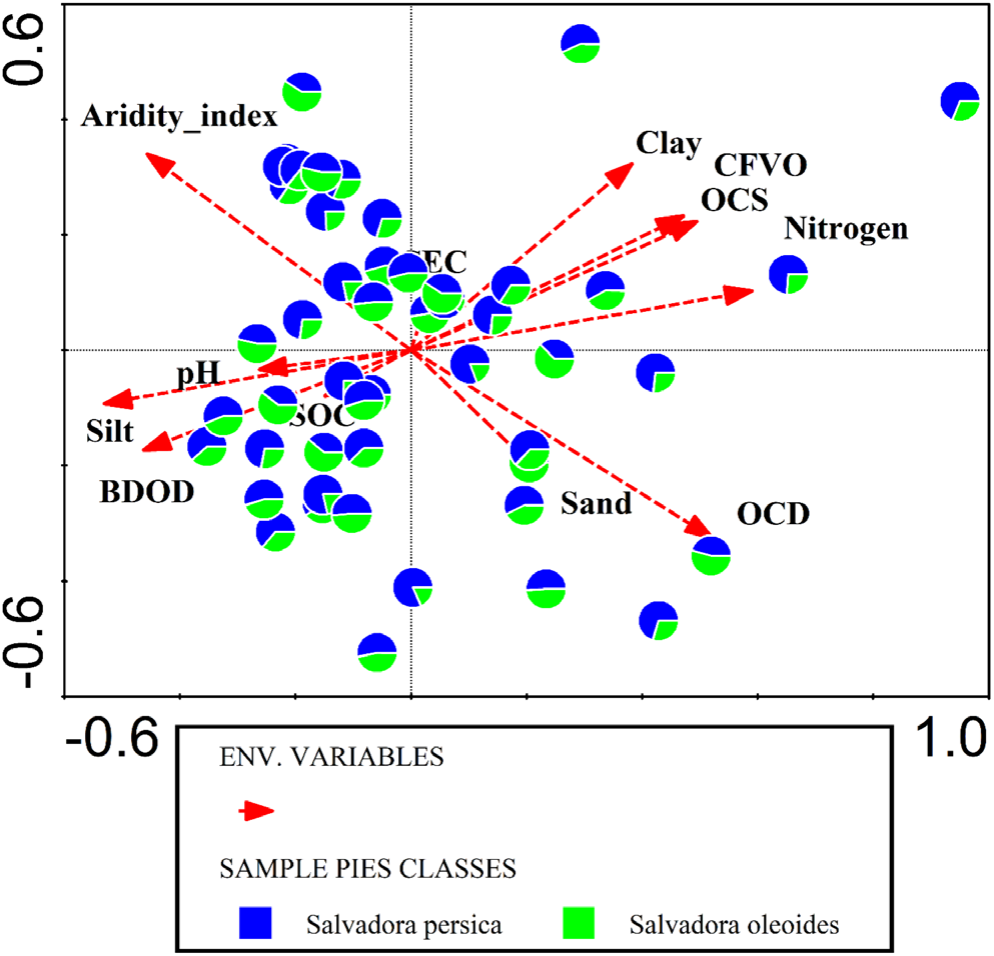
Canonical correspondence analysis (CCA) showing the impact of edaphic variables on the distribution of *Salvadora* species in semi‐arid regions.

A Canonical Correspondence Analysis (CCA) was performed to examine the impact of edaphic variables on the distribution of the genus *Salvadora*. The analysis revealed that the total variation explained by the model was apportioned across the first four axes with eigenvalues of 0.0013, 0.0008, 0.0004, and 0.0003, respectively. The cumulative explained variation was 9.9% for Axis 1, 15.66% for Axis 2, 18.98% for Axis 3, and 21.17% for Axis 4 (Table 1). The pseudo‐canonical correlations for these axes were 0.6896 for Axis 1, 0.5652 for Axis 2, 0.4799 for Axis 3, and 0.4835 for Axis 4, indicating moderate relationships between the environmental variables and the axes (Figure 8, Table 3). The cumulative explained fitted variation was 38.79%, 61.37%, 74.4%, and 82.97% for the respective axes. Further investigation into the simple term effects of individual edaphic variables highlighted several key factors. The aridity index accounted for the largest proportion of variation, explaining 26.7% with a pseudo‐*F* value of 3.5 and a statistically significant *p*‐value of 0.004. Soil pH also had a notable impact, explaining 16.4% of the variation, with a pseudo‐*F* value of 3.3 and a *p*‐value of 0.004. Sand content was another significant variable, explaining 14.8% of the variation with a pseudo‐*F* value of 2.4 and a *p*‐value of 0.026. Organic carbon density (OCD) contributed 12.1% of the explained variation, though it was marginally non‐significant (pseudo‐*F* = 2, *p* = 0.052). Other variables, such as nitrogen content (9.6%, pseudo‐*F* = 1.8, *p* = 0.07), coarse fragments by volume (CFVO, 8.6%, pseudo‐*F* = 1.3, *p* = 0.256), and organic carbon stock (OCS, 3.3%, pseudo‐*F* = 1.1, *p* = 0.32) had less significant impacts. Variables such as clay, silt, bulk density (BDOD), Cation Exchange Capacity (CEC), and Soil Organic Carbon (SOC) explained minimal variation and was not statistically significant, with *p*‐values well above the 0.05 threshold. CCA indicated that certain edaphic variables, particularly the aridity index, soil pH, and sand content, significantly influence the distribution of Salvadora (Table 3).

**TABLE 3:** Effect of each edaphic variable on the distribution of Salvadora in the semi‐arid region.

Variables	Explains %	pseudo‐*F*	*p*
Aridity index	26.7	3.5	0.004
pH	16.4	3.3	0.004
Sand	14.8	2.4	0.026
OCD	12.1	2	0.052
Nitrogen	9.6	1.8	0.07
CFVO	8.6	1.3	0.256
OCS	3.3	1.1	0.32
Silt	2.5	1	0.446
Clay	1.8	0.9	0.5
BDOD	1.7	0.8	0.61
CEC	1.4	0.5	0.824
SOC	1.1	0.5	0.82

## 4 Discussion

The results indicate a significant predominance of *S. persica* in most regions, with the notable exception of Sahiwal, where both species show similar densities. Thisregional variation indicates that the distribution and density of *Salvadora* species in the semi‐arid lowlands are significantly influenced by local environmental factors (Bhandari et al. 2021; Waheed et al. 2022). Soil composition, water availability, and microclimatic conditions are known to have a significant impact on species distribution in arid and semi‐arid environments, and they will probably be significant factors in this context as well (Wang et al. 2012). Additionally, differences in past land management and land use practices may also account for the observed variations in tree densities (Shao et al. 2022). The varying densities of *S. oleoides* and *S. persica* in different places indicate that more research is necessary to better understand the ecological preferences and adaptive strategies of these species.

According to our research, *S. persica* and S. *oleoides'* functional characteristics differ significantly among the various habitats in the Sahiwal Division, demonstrating how they have adapted to the local environment. We contrasted these results with research from other semi‐arid areas to put them in perspective. *S. oleoides* populations in Punjab, Pakistan's arid regions show a great deal of variation in structural traits like sclerification, epidermal thickness, and stomatal traits, all of which are critical for conserving water and adapting to harsh environmental conditions. In a similar vein, *S. persica* exhibits metabolic adaptations to drought stress, such as the buildup of antioxidative and osmoprotectant compounds, which aid in its survival in arid environments (Iqbal et al. 2021; Rangani et al. 2020).

Comparative research conducted in other semi‐arid areas, like Haryana, India, has demonstrated that tree and shrub species use functional leaf traits, such as increased specific leaf area and leaf dry matter content, to adapt to high temperatures and water scarcity. These adaptations indicate convergent evolutionary strategies among woody plants in semi‐arid ecosystems, which is in line with our observations of *Salvadora* species in the study area (Punia and Jakhar 2023). Additionally, studies on roadside vegetation in Punjab's semi‐arid regions show that native species have higher biomass of roots and stems, adaptations that increase their resistance to environments with limited water and nutrient availability. Our findings that *S. oleoides* and *S. persica* exhibit habitat‐specific differences in tree height and trunk diameter are consistent with this, demonstrating their ability to adapt their growth patterns to environmental factors (Arshad et al. 2024). These comparative studies demonstrate how *Salvadora* species can adapt to a variety of semi‐arid conditions and stress the significance of functional trait variability for plant survival and ecosystem health. Including these comparisons improves our comprehension of the ecological tactics these species use and guide conservation initiatives in arid and semi‐arid areas.

According to the study, *S. oleoides* and *S. persica* differ significantly in important functional attributes in a range of habitats, which reflects their capacity for adaptation to the environment. Whereas the smallest measurements were found along rail tracks, where higher disturbance and poorer soil environments predominate, *S. persica* tended to have larger trunk diameters and greater tree heights in roadside areas, probably because of more favorable growth (Phulwaria et al. 2011; Zhang and Jim 2014; Xie et al. 2021). There was a noticeable variation in the Specific Leaf Area (SLA) and Leaf Dry Matter Content (LDMC) as well; the roadside habitats had the highest values of both, suggesting improved photosynthetic efficiency and water retention (Khatri et al. 2022). While results show that roadside conditions are more favorable for growth, *S. oleoides* showed greater trunk diameters and tree heights in roadside environments compared to archaeological sites. Additionally, *S. oleoides's* SLA and LDMC differed depending on the habitat. The species' unique adaptations to various environmental factors were highlighted by higher SLA in graveyards and increased LDMC along railway lines (Tounekti, Al‐Turki, et al. 2017; Tounekti, Mahdi, and Nouiri 2017; Oktavia and Jin 2020).

These results demonstrate how *Salvadora* species have adapted to live in different environments, which is essential to their persistence and function in semi‐arid ecosystems. This is consistent with other research (Pérez‐Ramos et al. 2019; Assaeed et al. 2020) that shows tree species in these harsh environments have significant trait flexibility to maximize resources and increase their survival chance. Changes in tree height and trunk diameter among habitats indicate that these species are able to modify their growth patterns in response to variables such as soil nutrients, water availability, and degree of disturbance. In certain habitats, higher SLA values indicate an adaptation intended to maximize photosynthetic efficiency, whereas variations in LDMC indicate strategies for striking a balance between water retention and photosynthetic efficiency (Peguero‐Pina et al. 2020). Other semi‐arid tree species have shown similar patterns of trait variability, suggesting that many woody plants in these conditions need to adapt in specific ways to survive (Grossiord et al. 2017; Carvajal et al. 2017; Dai et al. 2020; Soheili et al. 2023). This study strengthens the body of evidence showing *Salvadora* species are extremely adaptive and can change their traits to survive in a range of surroundings.

According to Kirmer et al. (2008), functional traits are essential to an organism's ability to adapt to its surroundings. Assessing how these traits vary and evolve within a genus can provide important information about the fundamental causes of evolutionary shifts and the adaptive significance of particular traits (Aubin et al. 2016). This information is essential for developing successful conservation plans for tree species (Massante et al. 2023; de Oliveira et al. 2020).

Our research helps us better understand trait variation within the genus by demonstrating that *Salvadora* species exhibit significant divergence in the functional traits evaluated across various habitats. The evolution of these traits happened in a correlated way across different taxonomic levels, and for the most part, the differences between species were more noticeable than those within a species (Iqbal et al. 2022, 2023). It was evident that the evolution of particular functional characters in Salvadora was greatly influenced by the climate, while their distribution was greatly impacted by edaphic factors like soil pH and sand content (Iqbal et al. 2021). The species' distinct adaptations to Punjab, Pakistan's semi‐arid climate are demonstrated by the variation in the phylogenetic patterns linked to these traits (Asif et al. 2023; Patel and Parida 2023).

Similar to patterns observed in other species, the diversity of these traits suggests a trade‐off whereby traits evolve to improve the ecological (Maggio et al. (2000); Tounekti, Al‐Turki, et al. 2017; Tounekti, Mahdi, and Nouiri 2017; Upendra et al. 2017; Barman et al. 2018). Primary branching species are found at archaeological sites; their leaves are larger and have a dry tissue characteristic. In contrast, species that have adapted to new settings—like roadside vegetation—have larger, more compact leaves. While temperature and rainfall are significant climatic factors that shape these characteristics, our canonical correlation analysis (CCA) showed that soil properties like pH and sand will have a significant impact on the distribution and development of leaf characteristics in Salvador.

Soil and climatic factors appear to have an impact on the genus' adaptive divergence. However, no one climatic condition can be shown to be the only source of the observed changes in features, despite the fact that various environmental factors have a considerable impact. This suggests that a variety of factors, including unspecified ones, may have contributed to the development of certain features (Soliveres et al. 2014; Steane et al. 2017; Silva et al. 2019). According to research, conditions that require efficient water use and photosynthesis are linked to traits like higher specific leaf area and lower leaf dry matter content (Garnier et al. 2001; Feng et al. 2008). These traits might be an adaptive reaction to the various soil characteristics and atmospheric circumstances found in Pakistan's semi‐arid Punjab regions.

In contrast to the trunk and branch diameter pattern, *Salvadora* species' Specific Leaf Area (SLA) and leaf size demonstrated only weak relationships between and among environments. The findings of Liu et al. (2016), which demonstrate that these traits are connected to multiple aspects of plant performance under diverse environmental circumstances, are in line with this discovery. According to earlier studies, we also found that, throughout the genus, leaf area increases with maximum plant height (Liu et al. 2010; Tomlinson et al. 2013; Conti and Díaz 2013; Pollock et al. 2018).

The analysis of all *Salvadora* species revealed that leaf size interacted favorably with habitat features like cemeteries and archaeological sites and negatively with aridity. Larger leaf species are frequently found in less arid environments, which is in line with findings from other taxa (Wright et al. 2002; Wigley et al. 2016). Our findings indicate that there is little relationship between *Salvadora* species' leaf characteristics and climatic conditions.

This suggests that a variety of factors, such as soil characteristics and microhabitat conditions, are likely influencing trait development (Ge et al. 2019; Mugnai et al. 2021). Because of the genus's widespread geographical distribution, it is difficult to identify specific factors that influenced the development of traits in each *Salvadora* species.

This complexity emphasizes the need for additional research to identify the various environmental factors influencing this species' evolution of traits and adaptation. Our research highlights the complex interplay between geographic and climatic factors that determine how *Salvadora* species function. Local environmental variables like terrain and soil qualities may also be important in determining trait variation within and among populations, even if temperature and precipitation have a major impact. Future research should explore these interactions to better understand how plants adapt to environmental conditions.

## 5 Conclusion

The distribution and functional traits of the *Salvadora* species in the semi‐arid subtropical soils of Pakistan's Sahiwal Division are the main subjects of this comprehensive study. Observations indicate that *S. persica* is present in most of the tessellations; in Sahiwal, on the other hand, *S. oleoides* and *S. persica* show similar patterns. Different functional traits, like dry matter content, leaf size, tree height, and trunk diameter, vary depending on the ecosystem to which a species has adapted. Edaphic parameters such as soil pH and dry index have a significant impact on distribution patterns in Salvadora, as do climatic fluctuations, particularly those related to temperature and precipitation. Despite their importance to the ecosystem and the economy, Salvadoran species are threatened by overfishing, habitat loss, and climate change. These difficulties highlight the importance of environmentally responsible management practices and conservation efforts to protect these species in their natural habitats. Subsequent investigations must focus on long‐term planning and the development of comprehensive conservation initiatives that include community engagement and adaptive management approaches. Key conservation strategies for protecting *Salvadora* species include in situ conservation through the protection of natural habitats such as graveyards, archaeological sites, and sacred groves, which serve as semi‐protected refuges. Ex situ conservation approaches, such as seed banking, nursery propagation, and plantation programs, are also recommended to ensure long‐term survival, especially under changing climatic conditions. Promoting community‐based resource management, regulated harvesting, and awareness programs is vital to reduce anthropogenic pressure and to enhance regeneration. Furthermore, integrating *Salvadora* species into reforestation and agroforestry systems can support ecological restoration and sustainable land use in semi‐arid regions. These strategies must be supported by policy frameworks and ecological monitoring.

## Author Contributions

**Shaheena Umbreen:** formal analysis (lead), investigation (lead), methodology (lead), writing – original draft (equal). **Naila Mukhtar:** conceptualization (lead), data curation (equal), project administration (lead), supervision (equal), writing – review and editing (equal). **Nidaa Harun:** project administration (equal), supervision (equal), validation (lead), writing – review and editing (equal). **Sajid Ali:** resources (equal), software (lead), supervision (equal), writing – review and editing (equal).

## Conflicts of Interest

The authors declare no conflicts of interest.

## Supporting information


**Table S1:** List of climatic and edaphic variables with details used to distribute genus *Salvadora*.

## Data Availability

The data that supports the findings of this study are available in the Supporting Information of this article.
