# Supplementary material for: Effect of Environmental Drivers on Functional Traits of Salvadora Population in the Semi‐Arid Regions: A Case Study From Division Sahiwal Pakistan
Source: Ecol Evol. 2025 Sep 2;15(9):e71938. doi: 10.1002/ece3.71938 (PMC12404651; doi:10.1002/ece3.71938)
Supplement: Supplementary file 1 — Table S1: List of climatic and edaphic variables with details used to distribute genus Salvadora. [file ECE3-15-e71938-s001.docx]

**Supplementary table 1**. List of climatic and edaphic variables with details used to distribute genus *Salvadora*.

| **Code** | **Name of Variable & Description** | **Database** | **Resolution** | **Unit** |
| --- | --- | --- | --- | --- |
| **Climatic variables** | | | | |
| Bio1 | Annual Mean Temperature | WorldClim | 30 arc s | °C |
| Bio2 | Mean Diurnal Range | WorldClim | 30 arc s | °C |
| Bio3 | Isothermality (Bio2/Bio7) (×100) | WorldClim | 30 arc s | Percent |
| Bio4 | Temperature Seasonality (sd ×100) | WorldClim | 30 arc s | °C |
| Bio5 | Max. Temperature of Warmest Month | WorldClim | 30 arc s | °C |
| Bio6 | Min. Temperature of Coldest Month | WorldClim | 30 arc s | °C |
| Bio7 | Temperature Annual Range | WorldClim | 30 arc s | °C |
| Bio8 | Mean Temperature of Wettest Quarter | WorldClim | 30 arc s | °C |
| Bio9 | Mean Temperature of Driest Quarter | WorldClim | 30 arc s | °C |
| Bio10 | Mean Temperature of Warmest Quarter | WorldClim | 30 arc s | °C |
| Bio11 | Mean Temperature of Coldest Quarter | WorldClim | 30 arc s | °C |
| Bio12 | Annual Precipitation | WorldClim | 30 arc s | Mm |
| Bio13 | Precipitation of Wettest Month | WorldClim | 30 arc s | Mm |
| Bio14 | Precipitation of Driest Month | WorldClim | 30 arc s | Mm |
| Bio15 | Precipitation Seasonality (CV) | WorldClim | 30 arc s | Percent |
| Bio16 | Precipitation of Wettest Quarter | WorldClim | 30 arc s | Mm |
| Bio17 | Precipitation of Driest Quarter | WorldClim | 30 arc s | Mm |
| Bio18 | Precipitation of Warmest Quarter | WorldClim | 30 arc s | Mm |
| Bio19 | Precipitation of Coldest Quarter | WorldClim | 30 arc s | Mm |
| **Edaphic variables** | | | | |
| BDOD | Bulk Density | SoilGrids | 30 arc s | cg/cm3 |
| CEC | Cations Exchange Capacity (pH: 7) | SoilGrids | 30 arc s | mmol(c)/kg |
| CFVO | Volumetric fraction of coarse fragments (>2 mm) | SoilGrids | 30 arc s | cm3/dm3 |
| Clay | Clay Contents | SoilGrids | 30 arc s | g/kg |
| Nitrogen | Total Nitrogen | SoilGrids | 30 arc s | cg/kg |
| OCD | Organic Carbon Density | SoilGrids | 30 arc s | hg/dm3 |
| pH | Soil pH × 10 | SoilGrids | 30 arc s | Nil |
| Sand | Sand Contents | SoilGrids | 30 arc s | g/kg |
| Silt | Silt Contents | SoilGrids | 30 arc s | g/kg |
| SOC | Soil Organic Carbon | SoilGrids | 30 arc s | dg/kg |
| OCS | Organic carbon stocks | SoilGrids | 30 arc s | kg/m² |
